# Direct Interaction between TalinB and Rap1 is necessary for adhesion of *Dictyostelium* cells

**DOI:** 10.1186/s12860-015-0078-0

**Published:** 2016-01-07

**Authors:** Katarzyna Plak, Henderikus Pots, Peter J. M. Van Haastert, Arjan Kortholt

**Affiliations:** Department of Cell Biochemistry, University of Groningen, Nijenborgh 7, Groningen, AG 9747 The Netherlands; Current address: BIOTEC center, Technical University Dresden, Tatzberg 47/49, 01307 Dresden, Germany

**Keywords:** Rap, talin, *Dictyostelium*, Adhesion, Chemotaxis, Development

## Abstract

**Background:**

The small G-protein Rap1 is an important regulator of cellular adhesion in *Dictyostelium*, however so far the downstream signalling pathways for cell adhesion are not completely characterized. In mammalian cells talin is crucial for adhesion and Rap1 was shown to be a key regulator of talin signalling.

**Results:**

In a proteomic screen we identified TalinB as a potential Rap1 effector in *Dictyostelium*. In subsequent pull-down experiments we demonstrate that the Ras association (RA) domain of TalinB interacts specifically with active Rap1. Studies with a mutated RA domain revealed that the RA domain is essential for TalinB-Rap1 interaction, and that this interaction contributes to cell-substrate adhesion during single-celled growth and is crucial for cell-cell adhesion during multicellular development.

**Conclusions:**

*Dictyostelium* Rap1 directly binds to TalinB via the conserved RA domain. This interaction is critical for adhesion, which becomes essential for high adhesive force demanding processes, like morphogenesis during multicellular development of *Dictyostelium*. In mammalian cells the established Rap1-talin interaction is indirect and acts through the scaffold protein - RIAM. Interestingly, direct binding of mouse Rap1 to the RA domain of Talin1 has recently been demonstrated.

**Electronic supplementary material:**

The online version of this article (doi:10.1186/s12860-015-0078-0) contains supplementary material, which is available to authorized users.

## Background

Proper regulation of substrate adhesion and cell motility is crucial for maintaining many cellular processes, including immune responses, angiogenesis and hemostasis [1, 2]. Misregulation of adhesion has been linked to the progression of cancer and autoimmune diseases [1]. The small G-protein Rap is critically involved in the regulation of cellular adhesion in many model organisms [3]. Rap belongs to the Ras- subfamily of small G-proteins that switch between an inactive GDP bound and active GTP bound state [4]. In the amoeba *Dictyostelium discoideum* Rap1 appears to have an essential role and is involved in regulating a multitude of cellular functions among which cell shape regulation, chemotaxis, substrate adhesion, multicellular development and cytokinesis [5–10]. Despite the important role of *Dictyostelium* Rap1 in regulating adhesion [11], the underlying signalling pathways are not fully understood. So far only the Serine/Threonine Kinase Phg2 has been identified as a key component of the Rap1-mediated adhesion pathway [11].

In mammalian cells Rap has been extensively linked to integrin signalling; integrin activation depends on Rap activity [12]. Integrin proteins, which are transmembrane receptors that work as αβ heterodimers and link the extracellular matrix to signalling events inside the cell, are essential for the formation of adhesion complexes [13, 14]. Binding of the large protein talin to the cytoplasmic domain of β-integrins is the final and crucial step in integrin activation in leukocytes and platelets [15]. Activation of Rap allows for RIAM translocation to the sites of active Rap at the cell membrane [16]. RIAM functions as a scaffold protein for talin and stimulates formation of adhesion complexes and subsequent integrin activation [17, 18].

There are no *Dictyostelium* RIAM proteins [19], while the only *Dictyostelium* MRL (Mig10/Riam/Lamellipodin) family protein binds to Ras and not to active Rap1 [20]. To identify potential Rap1 effectors that regulate adhesion we performed a proteomic screen of Rap1 interactors and found TalinB. *Dictyostelium*, similar to vertebrates, possesses two talin homologues [21]. While deletion of either *talinA* or *talinB* does not strongly affect substrate adhesion of vegetative cells, deletion of both *talinA* and *talinB* genes results in cells that are incapable of adhering to substrates [22]. During the *Dictyostelium* developmental cycle, which is initiated by starvation, single cells form large multicellular aggregates that by primitive morphogenesis develop into fruiting bodies [23]. TalinB is crucial for morphogenesis as it appears to mediate the transmission of force from the molecular motors inside the cell to the extracellular environment, which provides traction needed for movement in the multicellular structures [24]. Lack of both TalinA and B homologues causes even stronger developmental defects as cells are incapable of forming tight multicellular aggregates, likely due to strong defects in cellular adhesion [22]. Importantly both substrate adhesion [15] and morphogenetic defects [25] are also associated with talin mutants in higher eukaryotic systems.

We show here that *Dictyostelium* Rap1 directly interacts with the RA domain of TalinB. Importantly, this binding of TalinB to activated Rap1 is essential for talin-mediated adhesion during *Dictyostelium* morphogenesis.

## Results and discussion

### Dictyostelium TalinB is a downstream effector of Rap1

**Fig. 1:**
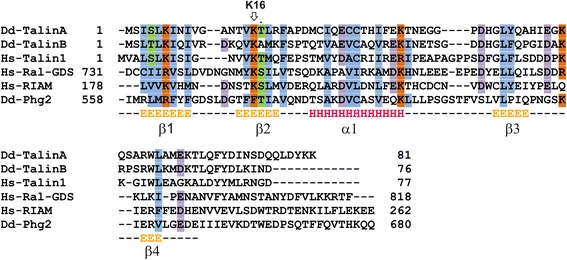
N-terminal part of *Dictyostelium* TalinB has a putative RA domain. Alignment of the putative RA domain of *Dictyostelium* TalinA and TalinB against the RA domains of human Talin1, Ral-GDS, RIAM and *Dictyostelium* Phg2. The conserved lysine K16 that is mutated in GFP-TalB-K16A is indicated. Structural elements are indicated below for β-sheets (E) and α-helix (H)

We recently used a proteomic approach to identify new components of the *Dictyostelium* Rap1 signalling pathways: Purified Rap1 loaded with a non-hydrolysable GTP analogue was used as a bait in a large scale pull-down from *Dictyostelium* cell lysates and the interactome was subsequently analysed by LC-MS [26]. A number of identified proteins, previously connected to processes related to cell migration, actin filament formation and multicellular development were identified in this screen (Additional file 1: Table S1). Among these, TalinB was identified with fourteen unique and specific peptides, which were not present in control samples (pull down with GST protein). The amino acid sequence of *Dictyostelium* TalinB and mouse Talin1 show conservation (27 % sequence identity and 47 % sequence similarity), especially in the head domain (45 % sequence identity and 64 % similarity) and the C-terminal I/LWEQ domain (39 % identity, 62 % similarity). Importantly, more detailed sequence alignments revealed that, like murine Talin1 [27], *Dictyostelium* TalinB contains a RA-like region in the F0 part of the FERM domain. Although there is generally no high sequence conservation between different RA domains [28], the F0 region of murine Talin1 (residues 1–85) showed 39 % sequence identity and 58 % sequence similarity to analogous sequence of *Dictyostelium* TalinB (Fig. 1).

**Fig. 2:**
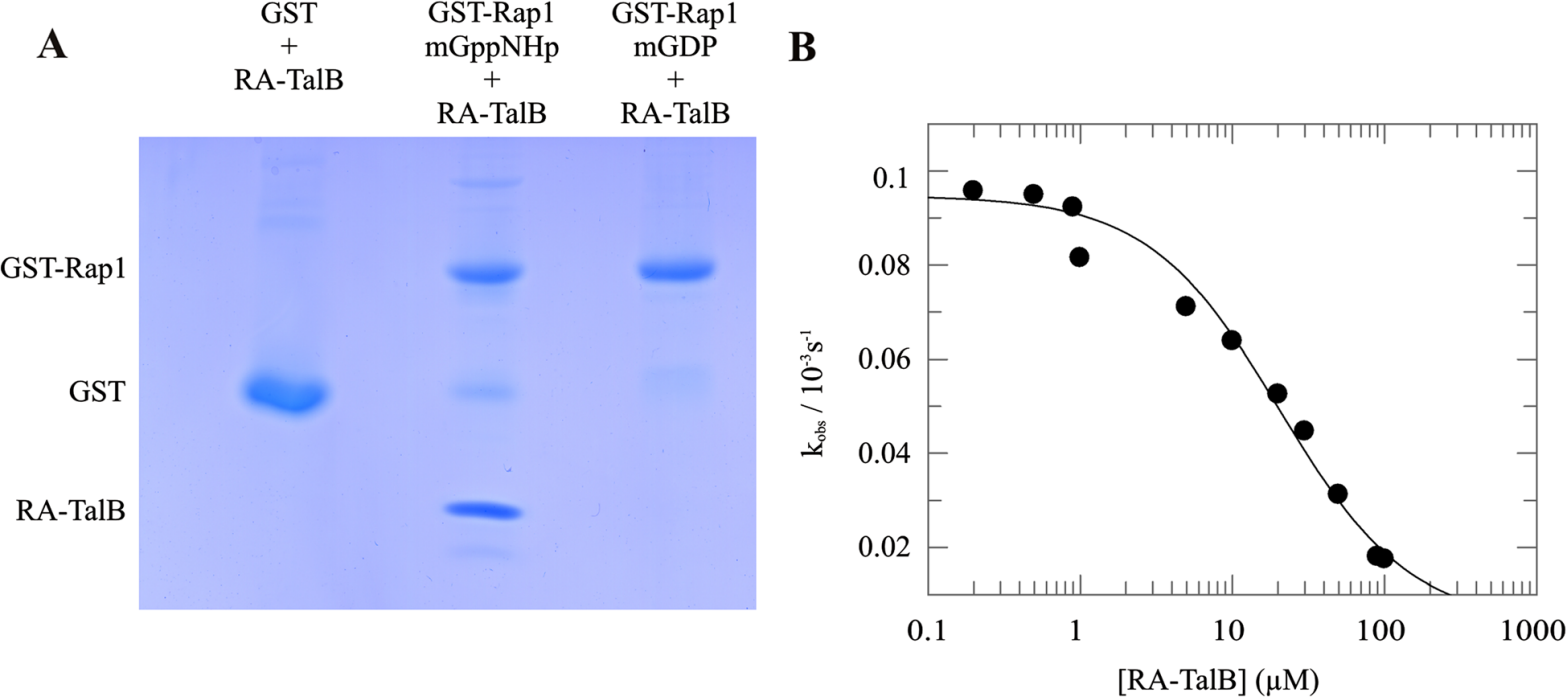
The Ra domain of TalinB interacts with Rap1 in a nucleotide dependent manner. **a** Pull down experiment with active and inactive GST-Rap1 protein as bait and purified RA domain of TalinB (RA-TalB) as prey. RA-TalB is precipitated with activated Rap1 but not with inactive Rap1 or GST control. **b** The dissociation rate of mGppNHp from Rap1 was measured in the presence of varying concentration of RA-TalB. These data were used to calculate the observed rate constants, k_obs_. The k_obs_ values were plotted against the indicated effector concentration of RA-TalB. The addition of increasing concentrations of the effector results in a concentration dependent decrease of k_obs_. These data were used to calculate the dissociation constant of the Rap1GTP/ RA-TalB

To assess if the RA domain of TalinB can directly bind to active Rap1 we performed *in vitro* binding studies. The *Dictyostelium* RA-TalB domain (residues 1–122) was purified as a GST- fusion protein from *E.coli* lysates by subsequent steps of affinity and size exclusion chromatography. This was followed by GST tag removal by TEV protease digestion. The obtained RA-TalB protein was used as prey in co-immunoprecipitation experiments with c-truncated recombinant GST-Rap1 fusion protein as bait. Rap1 was preloaded with GppNHp (active) or GDP (inactive) and incubated with GSH-beads in the presence or absence of RA-TalB protein. The RA domain of TalinB interacts with GppNHp loaded GST-Rap1 protein, but not with inactive GST-Rap1-GDP or GST alone (Fig. 2a). To further confirm and characterize the TalinB/Rap interaction *in vitro* GDI (Guanine nucleotide Dissociation Inhibition) assays were performed [29]. The assay is based on the general observation that effector binding to small G-proteins stabilizes nucleotide binding to the G-protein; therefore effectors of small G-proteins inhibit the dissociation of nucleotide from that G-protein [29]. Incubating Rap1 preloaded with the fluorescent nucleotide analogue (mGppNHp) with an excess of non-fluorescent GppNHp results in nucleotide exchange that can be measured by a decay in fluorescence, from which the rate constant k_obs_ is calculated. The addition of increasing concentrations of TalB-RA results in a concentration dependent decrease of k_obs_ (Fig. 2b). The affinity (K_d_) between Rap1 and TalB-RA was determined from this dependency, yielding a K_d_ of 19.9 +/- 4.8 μM. In contrast, no inhibition of nucleotide exchange was observed when mGppNHp loaded RasG was used (Additional file 2: Figure S1). Together, the data show that TalinB specifically interacts with active Rap1.

### TalinB - Rap1 interaction is essential for multicellular development of Dictyostelium cells

**Fig. 3:**
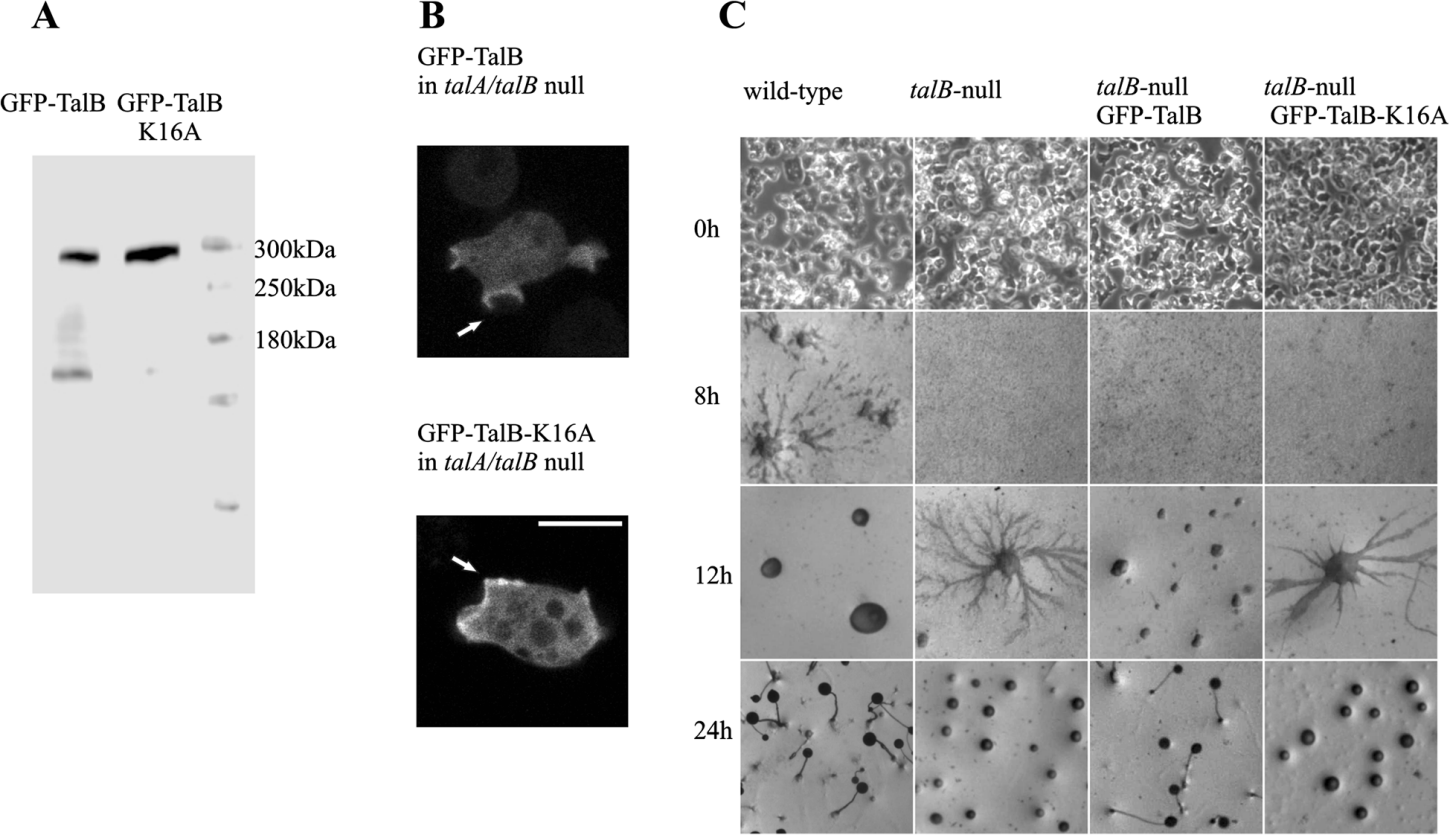
A TalinB mutant that does not interact with Rap1 cannot rescue development *of talB*-null cells. **a** Western blot with α-GFP antibody on *Dictyostelium talA/B*-null cell lysate expressing GFP-TalB or GFP-TalB-K16A. **b** Mutations in the RA domain of TalinB do not influence protein localization. Wild-type GFP-TalB and mutated GFP-TalB-K16A were overexpressed in *talA/talB-*null background and their localization was tested using confocal microscopy. Scale bar represents 10 μm. **c** Wild-type cells, *talB-*null cells and *talB*-null cells expressing wild-type GFP-TalB or mutated GFP-TalB-K16A were allowed to develop at non nutrient agar for 30 h; development of the cells was monitored by light microscopy. As previously shown *talB-*null cells are arrested in development at the mound phase. This defect cannot be rescued by the GFP-TalB-K16A mutant.

*In silico* analysis and crystallographic studies have shown that the interaction between small G-proteins and RA domains are mediated by exposed charged residues in the three secondary structure elements, β1, β2 and α1 of the ubiquitin like fold of the RA domains (Fig. 1, [28, 30].) Amino acid K16 of TalinB is conserved in almost all RA domains and predicted to be one of the important positively charged amino acids for the RA/small G-protein interaction (Fig. 1) [30]. To study the biological relevance of the Rap1 and TalinB interaction in more detail, we generated and expressed a full-length TalinB-K16A mutant fused to GFP in *talA/talB*-null cells. Western blot analysis showed that both GFP-TalB wild-type and the GFP-TalB-K16A mutant proteins are expressed at similar levels in *talA/talB-*null cells (Fig. 3a), however a short degradation product is visible for the GFP-TalB wild-type protein. Confocal microscopy revealed that both wild-type GFP-TalB and mutant GFP-TalB-K16A are localized at the plasma membrane in cups (Fig. 3b). The experiments suggest that GFP-TalB-K16A is properly expressed and folded in *Dictyostelium* cells.

Upon starvation *Dictyostelium* cells enter multicellular development stage which after 24 h results in the formation of fruiting bodies carrying spores of *Dictyostelium* cells (Fig. 3c). At the late stages of development TalinB is necessary for the cells to migrate within the multicellular structures, a process that depends on adhesion and force transmission between the cytoskeleton and cell exterior [24]. Cells lacking *talB* are able to initiate starvation and form large aggregates, but the process stops at this mound stage and cells are unable to culminate and produce fruiting bodies [31] (Fig. 3c). Re-expression of GFP-TalB in *talB*-null cells completely rescues the developmental defect and cells are able to produce normal fruiting bodies (Fig. 3c). In contrast, *talB*-null cells expressing GFP-TalB-K16A are unable to culminate and produce spores, suggesting that TalinB-Rap1 interaction is essential for these processes.

### TalinA and TalinB dependent Rap1 adhesion in vegetative cells

**Fig. 4:**
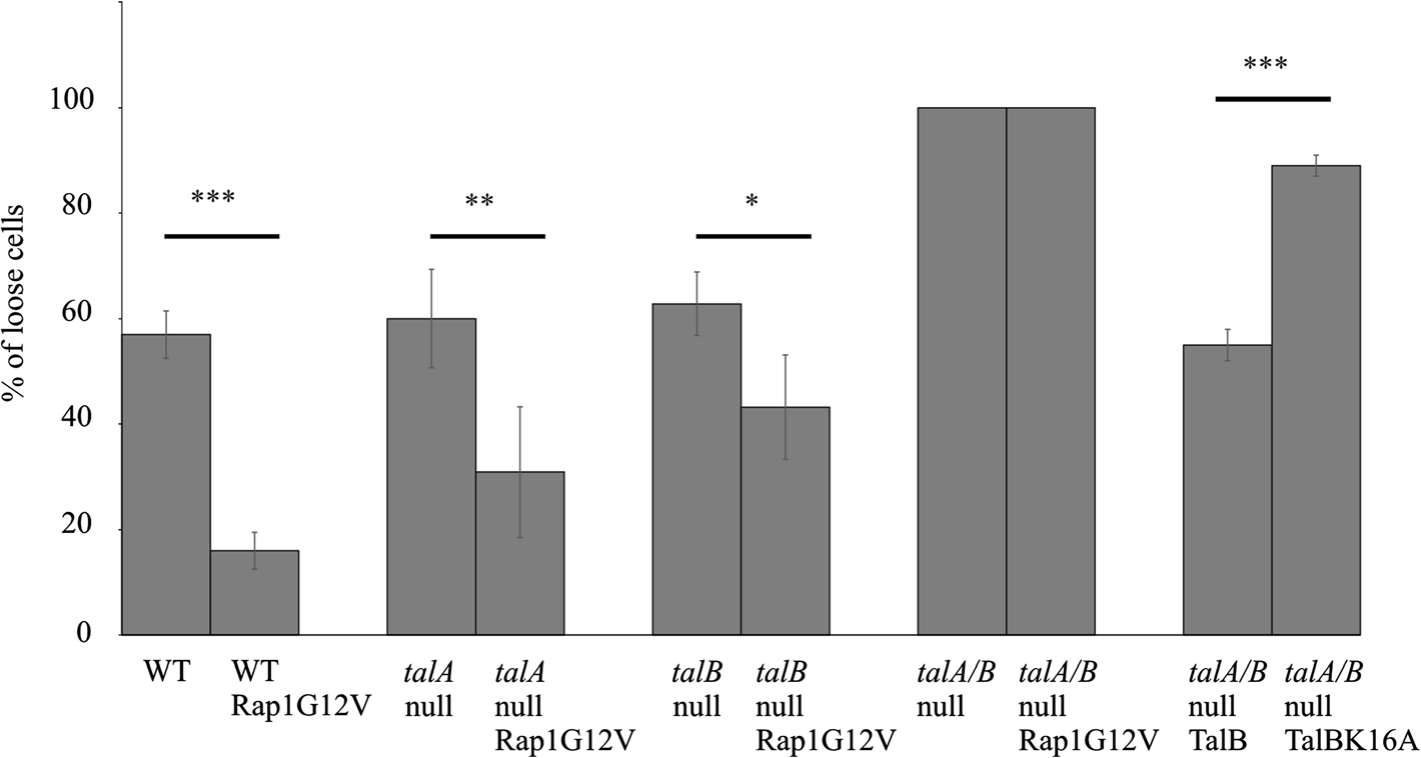
Adhesion of wild type and talin mutant cells to substratum. Cells were allowed to adhere to the plastic surface of a Petri dish and then shaken for 1 h at 150 rpm. Shown is the percentage of cells that do not adhere to the plate. Presented are the means and SD of four independent experiments. * indicates *P* < 0.05; ** indicates *P* < 0.01; *** indicates *P* < 0.0001 as determined by student *t*-test

The strength of substrate adhesion of vegetative cells can be measured by a plate shaking assay [32], where cells are incubated on a rotary shaker for an hour and the amount of attached and detached cells are calculated in triplicates The percentage of loose cells after one hour of shaking was not significantly different for *talA*-null (60.0 ± 9.3 %) and, *talB*-null (62.8 ± 6 %) compared to the wild-type strain (57.0 ± 4.5 %) (Fig. 4). This is consistent with previous studies that showed that either TalinA or TalinB is sufficient for mediating *Dictyostelium* adhesion [22]. Deletion of both *talA* and *talB* results in cells that are incapable of adhering to substrates (100 % loose cells). Consistently, with a previous report [22], this defect in adhesion does not dependent on the rotation speed, as even cells that were kept stationary were unable to attach to substrates. Expression of wild-type TalinB in *talA/talB*-null cells resulted in adherent cells (55 % loose cells) similar to what is observed for *talA*-null and wild-type cells. In contrast, expression of the RA mutant TalB-K16A in *talA/talB*-null cells resulted in only slightly improved cellular adhesion (89 ± 2 % loose cells) as compared to *talA/talB-*null cells. These TalB-K16A expressing cells are more adhesive than the 100 % loose parental *talA/talB*-null strain, but much less adhesive when compared to the 55 % loose cells of wild-type TalinB in *talA/talB*-null cells. Together the results suggest that direct binding of Rap1 to the RA domain of TalinB is important for TalinB to mediate major cell attachment.

We further used the inducible expression system [33] to investigate the effects of hyperactivated *Dictyostelium* Rap1 (Rap1G12V) on talin mediated adhesion. As reported before, expression of Rap1G12V in wild-type cells results in a strong increase of cell-substrate adhesion ([5], Fig. 4). In contrast, expression of Rap1G12V in *talA/talB*-null cells has no effect on the adhesion properties of this stain. Both the uninduced *talA/talB*-null control as well as the induced Rap1G12V expressing cells showed the same characteristic phenotype; cells float in the growth medium and do not adhere to the substratum. This strongly suggests that the presence of talin is crucial for Rap1 mediated cellular adhesion.

To address if Rap-mediated increased adhesion depends on TalinA, TalinB or both, we expressed Rap1G12V in the *talA*-null or *talB*-null strains. Wild-type cells expressing Rap1G12V show within 24 h of induction a strong flattening of the cells which is associated with strongly increased cellular adhesion (16.0 ± 3.5 % loose cells). Interestingly, expression of Rap1G12V in *talA*-null or *talB*-null cells had a significantly smaller effect on cell adhesion, resulting in 30.9 ± 12.4 % and 43.2 ± 9.9 % of loose cells, respectively (Fig. 4). Together the results show that Rap1 can regulate the strength of cellular adhesion via both TalinA and TalinB.

## Conclusions

**Fig. 5:**
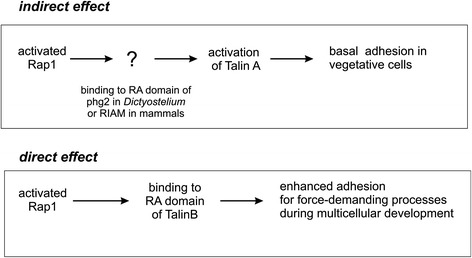
Model for the direct and indirect interaction of Rap1 with talin in *Dictyostelium* and mammalian cells. Rap-mediated activation of TalinA induces basal adhesion, possibly through binding to the RA domain of Phg2 in *Dictyostelium* or RIAM in mammalian cells. Activated Rap can also bind directly to the RA domain of TalinB, thereby inducing additional adhesion for force-demanding processes

Cellular adhesion is important during both the vegetative state as well as during multicellular development of *Dictyostelium* cells. We showed that in vegetative cells Rap1-mediated adhesion depends on the presence of either TalinA or TalinB. Our studies further showed direct binding interaction between TalinB and Rap1, and the experiments with a TalinB mutant that is incapable of binding Rap1 revealed strongly diminished adhesion. Moreover, we observed that overexpression of the dominant active Rap1G12V in wild-type cells leads to strongly increased adhesion, whereas overexpression of Rap1G12V in *talB*-null cells leads to only a mild increase of adhesion. These data strongly suggest that Rap1 induces cell adhesion through direct binding to the RA domain of TalinB (Fig. 5). As shown by the developmental defect of *talB*-null cells, this interaction is especially important for processes that require strong adhesive forces such as during erection of the sorocarp. Binding of small G-proteins to effectors can induce recruitment of the protein to the plasma membrane and/or activation of the protein [34]. We observed that both wild-type TalinB and TalinB-K16A are localized at the cups on plasma membrane (Fig. 3c), indicating that the localization of TalinB at the plasma membrane does not depend on its ability to bind Rap1. This strongly suggest that Rap1 regulates TalinB signalling by local allosteric activation rather than by recruitment of the protein. Such an allosteric mechanism of activation also has been proposed for the *Dictyostelium* Rap effector Phg2, but differs from the human Rap effector RIAM and Ras-regulated kinases such as Raf-1 that regulate effector recruitment to the membrane rather than its direct activation [11, 17, 34].

Several observations suggest that not all Rap1-mediated adhesion is exclusively mediated by TalinB. First, although the TalinB-K16A mutant can no longer bind to Rap1 and has strongly diminished adhesion relative to wild type TalinB (89 % of TalinB-K16A in *talA/talB*-null compared to 55 % loose cells of TalinB in *talA/talB*-null), its 89 % loose cells is significantly different from to 100 % loose cells of *talA/talB*-null cells. Moreover, overexpression of dominant active Rap1G12V in *talB*-null cells lead to enhanced adhesion, although not as strong as in wild-type cells overexpressing Rap1G12V. Both observations suggest that Rap1 mediates partial adhesion in the absence of TalinB. Since Rap1G12V does not induce adhesion in *talA/talB*-null cells, it is likely that the Rap1-induced TalB-independent adhesion is mediated by TalinA. The phenotype of Rap1G12V in *talA*-null cells suggest that TalinA could indeed be responsible for Rap1-mediated adhesion in vegetative cells. Since we so far were unable to purify sufficient amounts of the TalinA RA domain for *in vitro* studies, we cannot exclude that active Rap1 may bind to the head domain of TalinA. However we were unable to detect binding of RapA to TalinA in *Dictyostelium* cell lysate. Alternatively, the *Dictyostelium* Rap1 effector Phg2 could be a key link in this pathway; Rap1-mediated adhesion in vegetative cells depends on Phg2 and cells lacking *phg2* have reduced TalinA levels [11, 35]. Whether TalinA and Phg2 are direct interaction partners remains unknown so far [35].

Also in mammalian cells Rap may stimulate talin-mediated adhesion through two pathways with direct and indirect activation of talin isoforms, respectively. RIAM is a scaffold protein for talin. Activation of Rap induces RIAM translocation to the sites of active Rap at the cell membrane where it stimulates Talin1 [16, 18] by preventing the formation of auto-inhibitory talin fold [17]. As in *Dictyostelium* this indirect pathway is responsible for the basal substratum adhesion (Fig. 5). Mammalian Talin1 might also be involved in a direct pathway, since structural and biochemical studies with mouse Talin1 revealed the presence of an RA like domain that specifically interacts with active Rap. Although the observed binding affinity of Rap and Talin1 was rather low and the biological significance of this interaction has not been shown yet [27], it is tempting to speculate, that in analogy to *Dictyostelium*, this direct pathway is involved in multicellular processes that require strong adhesion forces such as morphogenesis.

## Methods

### Strains and cell cultivation

*Dictyostelium talA*-null cells were obtained from the *Dictyostelium* stock center [19], and *talB*-null and *talA/talB*-null cells were generated by Dr. Masatsune Tsujioka and kindly provided by Dr. Taro Uyeda and the National Institute of Advanced Industrial Science and Technology (AIST) Japan. Cells were grown on nunclon coated plates or in erlenmeyer flasks in HL5-C media (Formedium) supplemented with the appropriate antibiotics: 10 μg/ml Blasticidin S, 10 μg/ml Geneticin, or 50 μg/ml Hygromycin B.

### Construction of the overexpressor plasmids

GFP-TalinB overexpressing construct was created by PCR of the TalinB coding sequence and the product was ligated to pDM317 *Dictyostelium* plasmid [36]. Plasmids with TalinBK16A mutations were created by quick-change PCR using pDM317*talb* plasmid as template and the primers F5′GATAAACAAGTTGCAGCAATGAAGTTCTC3′ and R5′CATTGCTGCAACTTGTTTATCTCTAACG3′. The RA domain construct (1–122) was created by PCR from a full length *talB* construct, digested by BamHI and ligated to pGEX4T-3 vector (GE Healthcare). All constructs were sequenced and the expression of proteins in *Dictyostelium* was further confirmed by means of Western Blot with anti-GFP antibody (sc-9996, Santa Cruz).

### Protein purification

C-truncated GST-Rap1 protein was purified as described previously [11], GST-RA-TalB protein (AA 1-122) was expressed in *E. coli* Rosetta cells. Cells were grown in TB media (Carl Roth) to an OD600 of 0.6, protein expression was induced with 0,1 mM IPTG (Isopropyl-1-thio-β-D-galactopyranoside) and incubated overnight at 22 °C. Subsequently, cells were harvested, and lysed in buffer containing 50 mM Hepes, 50 mM NaCl, 5 mM DTT, 5 mM MgCl_2_, pH 7,5, and Proteinase inhibitor mix (60 μg/ml N-CBZPA, 100 μg/ml TAME, 80 μg/ml TPCK, 2 μg/m Pepstatin and 5 μg/ml Leupeptin). Cells were lysed by sonication, the lysate was cleared by centrifugation (75600 g, 4 °C, 45 min) and purified using a Glutathione Sepharose (GSH) - affinity column (GE-Healthcare). GST-RA-TalB was eluted in buffer containing 50 mM Hepes, 50 mM NaCl, 5 mM DTT, 5 mM MgCl_2_ and 20 mM Glutathione, pH7,5. The GST tag was cleaved by TEV protease digestion (0.06mgTEV/1mG-protein) overnight at 4 °C. Free GST was removed using a GSH column and the RA-TalB protein was further purified by size exclusion chromatography (Sephacryl 16/60, GE Healthcare). The isolated protein was analysed using SDS-page and the protein concentration was determined using the method of Bradford (Biorad).

### Pull down experiment

GST-Rap1 was loaded with GppNHp or GDP by incubating it in the presence of 10 mM EDTA and a 20-fold excess of the nucleotide for 2 h at room temperature [11]. Nucleotide binding to the G-proteins was stabilized by addition of MgCl_2_ to a final concentration of 20 mM. The G-proteins were prebound to GSH beads (GE Healthcare) by incubation for 2 h at 4 °C. The G-protein-coupled beads were washed with assay Buffer (50 mM Tris pH 7,5, 50 mM NaCl, 5 mM DTT, 10 mM MgCl_2_) and subsequently incubated with 50 μg of RA-TalB protein for 2 h at 4 °C. The GSH beads were harvested by centrifugation and washed three times in assay buffer. The samples were boiled in 1xSDS loading buffer and subjected to SDS-page.

### GDI assays

GDI assays were performed as described previously [29]. Shortly, mGppNHp labelled Rap1 or RasG were incubated at 25 °C in assay buffer (50 mM Tris–HCl, pH 7.5, 5 mM MgCl_2_, 50 mM NaCl and 5 mM DTE) in the presence or absence of the indicated amounts of RA-TalB. The reaction was started by the addition of 200 fold excess of GppNHp and the nucleotide release was recorded as a decrease in fluorescence intensity using a Spex spectrofluorometer (Spex Industries).

### Adhesion assays

To quantify the attachment to the substratum we used a previous described protocol [32]. Briefly, *Dictyostelium* cells were grown on coated nunclon dishes to a maximum of 70 % confluence. Floating cells were removed and fresh media was supplemented to the cells. Samples were placed on a rotary shaker for 1 h at 250 rpm. Subsequently, the number of loose and adhesive cells was determined in triplicate in Thoma counting chambers. The data is represented as an average of four experiments each performed on different day.

### Microscopy

*In vivo* localization of GFP-TalinB was monitored using a LSM 510 META-NLO microscope (Carl Zeiss Microimaging, Inc) equipped with a 63×/NA 1.4 objective (Plan-Apochromatic; Carl Zeiss Microimaging, Inc.) GFP (S65T) fluorochrome was excited with 488-nm argon/krypton laser and the emission was filtered through a BP500-530 IR filter and detected by a photomultiplier tube. Cell starvation and development was assayed by washing *Dictyostelium* cells in PB buffer (10 mM KH_2_PO_4_/Na_2_HPO_4_, pH 6.5) and plating out on layers of non-nutrient agar (1,5 % agar in PB). The pictures were registered using a digital camera (ScopeTek DCM130) connected to stereo microscope.

### Availability of supporting data

All the supporting data are included as additional files.

## Additional files

Additional file 1: Table S1. A subset of proteins identified in Mass Spectrometry analysis of Rap1 pull down sample. Proteins have been identified as present in the sample if a minimum of 2 unique peptides were identified in Rap1 pull down sample that were not present in the GST control sample. (DOCX 22 kb) Additional file 2: Figure S1. Dissociation of mGppNHp from RasG in the absence and presence of 100 μM purified RA-TalB. (TIF 430 kb)

## Abbreviations

DTT: Dithiothreitol
GDI: Guanine nucleotide Dissociation Inhibition
GDP: Guanosine diphosphate
GppNHp: Guanosine-5′-[βγ-imido]triphosphate
GTP: Guanine triphosphate
GSH: Glutathione Sepharose
GST: Glutathione S-transferase
IPTG: Isopropyl-1-thio-β-D-galactopyranoside
mGppNHp: 2′/3′-O-(N-Methyl-anthraniloyl)-guanosine-5′-(βγ)-imido]triphosphate
RA: Ras Association
TEV: Tobacco Etch Virus
