# Supplementary material for: Direct Interaction between TalinB and Rap1 is necessary for adhesion of Dictyostelium cells
Source: BMC Cell Biol. 2016 Jan 7;17:1. doi: 10.1186/s12860-015-0078-0 (PMC4861126; doi:10.1186/s12860-015-0078-0)
Supplement: Additional file 1: Table S1. — A subset of proteins identified in Mass Spectrometry analysis of Rap1 pull down sample. Proteins have been identified as present in the sample if a minimum of 2 unique peptides were identified in Rap1 pull down sample that were not present in the GST control sample. (DOCX 22 kb) [file 12860_2015_78_MOESM1_ESM.docx]

| \| Protein ID \| Protein Name \| Unique peptides  Rap1 \| Unique peptides  Control \| Related GO terms \| \| --- \| --- \| --- \| --- \| --- \| \| Q54K81 \| talB \| 19 \| 5 \| actin cytoskeleton (GO:0015629), cell-substrate adhesion (GO:0031589), sorocarp development (GO:0030587) \| \| P27133 \| corA \| 12 \| 0 \| negative regulation of actin filament depolymerisation (GO:0030835), regulation of myosin II filament disassembly (GO:0043521), aggregation involved in sorocarp development (GO:0031152), endocytosis (GO:0006897), phagocytosis (GO:0006909), macropinocytosis (GO:0044351) \| \| P42528 \| arpC \| 3 \| 1 \| Arp2/3 protein complex (GO:0005885), positive regulation of actin filament polymerisation (GO:0030838), phagocytosis (GO:0006909), macropinocytosis (GO:0044351) \| \| P54686 \| aip1 \| 11 \| 3 \| cortical actin cytoskeleton (GO:0030864), phagocytosis (GO:0006909), pinocytosis (GO:0006907) \| \| Q54HG2 \| ctxA \| 9 \| 0 \| actomyosin contractile ring (GO:0005826), regulation of myosin II filament assembly (GO:0043520), epithelial cell morphogenesis (GO:0003382), aggregation involved in sorocarp development (GO:0031152), sorocarp stalk morphogenesis (GO:0036360) \| \| Q54P24 \| gxcC \| 15 \| 0 \| Rho guanyl-nucleotide exchange factor activity (GO:0005089) \| \| Q54RV9 \| sglA \| 4 \| 0 \| regulation of actin filament polymerization (GO:0030833), sporulation resulting in formation of a cellular spore (GO:0030435), sorocarp development (GO:0030587), negative regulation of aggregate size involved in sorocarp development (GO:0031158) \| \| Q54TU2 \| vinB \| 43 \| 7 \| actin cytoskeleton (GO:0015629), cell adhesion (GO:0007155) \| \| Q550R2 \| ctxB \| 7 \| 0 \| regulation of myosin II filament assembly (GO:0043520), cell morphogenesis (GO:0000902), sorocarp development (GO:0030587), aggregation involved in sorocarp development (GO:0031152) \| \| Q869Q3 \| napA \| 9 \| 1 \| positive regulation of actin filament polymerization (GO:0030838), cell adhesion (GO:0007155), single organismal cell-cell adhesion (GO:0016337), cell-substrate adhesion (GO:0031589) \| \| Q86L04 \| trap1 \| 6 \| 0 \| cortical actin cytoskeleton (GO:0030864), multicellular organismal development (GO:0007275), cell differentiation (GO:0030154), sporulation resulting in formation of a cellular spore (GO:0030435) \| \| P15064 \| rasG \| 7 \| 1 \| cell-substrate adhesion, regulation of calcium-dependent cell-cell adhesion, cell morphogenesis (GO:0000902), aggregation involved in sorocarp development (GO:0031152) \| \| Q55FS2 \| krsB \| 5 \| 0 \| cell adhesion (GO:0007155) \| \| P25870 \| chcA \| 17 \| 0 \| sporulation resulting in formation of a cellular spore (GO:0030435), aggregation involved in sorocarp development (GO:0031152) \| \| P54654 \| cap \| 13 \| 0 \| cell morphogenesis (GO:0000902) \| \| Q54BM7 \| ppkA \| 2 \| 0 \| sporulation resulting in formation of a cellular spore (GO:0030435), phagocytosis (GO:0006909), macropinocytosis (GO:0044351) \| \| Q54K32 \| rgaA \| 7 \| 1 \| epithelial cell morphogenesis (GO:0003382), sporulation resulting in formation of a cellular spore (GO:0030435), sorocarp development (GO:0031152),sorocarp stalk cell differentiation GO:0031149, culmination involved in sorocarp development (GO:0031154), sorocarp stalk morphogenesis (GO:0036360) \| \| Q55BM4 \| tcp1 \| 12 \| 0 \| sorocarp development (GO:0031152) \| \| Q55BV5 \| psmC1 \| 6 \| 0 \| sorocarp development (GO:0031152) \| \| Q8MQU6 \| cshA \| 8 \| 0 \| sorocarp morphogenesis (GO:0031288), phagocytosis (GO:0006909), macropinocytosis (GO:0044351) \| \| Q94502 \| modA \| 5 \| 0 \| sorocarp morphogenesis (GO:0031288) \| \| Q9NKW1 \| mfeA \| 2 \| 0 \| sorocarp development (GO:0031152) \| \| Q9XZJ3 \| culB \| 5 \| 0 \| multicellular organismal development (GO:0007275), negative regulation of sorocarp stalk cell differentiation (GO:0031286) \| \| Q23858 \| crtA \| 12 \| 3 \| Phagocytosis (GO:0006909), engulfment (GO:0006911), macropinocytosis (GO:0044351) \| \| Q02158 \| plc \| 4 \| 0 \| Lipid metabolic process (GO:0006629) \| \|  \|  \|  \|  \|  \| |
| --- | --- | --- | --- | --- | --- | --- | --- | --- | --- | --- | --- | --- | --- | --- | --- | --- | --- | --- | --- | --- | --- | --- | --- | --- | --- | --- | --- | --- | --- | --- | --- | --- | --- | --- | --- | --- | --- | --- | --- | --- | --- | --- | --- | --- | --- | --- | --- | --- | --- | --- | --- | --- | --- | --- | --- | --- | --- | --- | --- | --- | --- | --- | --- | --- | --- | --- | --- | --- | --- | --- | --- | --- | --- | --- | --- | --- | --- | --- | --- | --- | --- | --- | --- | --- | --- | --- | --- | --- | --- | --- | --- | --- | --- | --- | --- | --- | --- | --- | --- | --- | --- | --- | --- | --- | --- | --- | --- | --- | --- | --- | --- | --- | --- | --- | --- | --- | --- | --- | --- | --- | --- | --- | --- | --- | --- | --- | --- | --- | --- | --- | --- | --- | --- | --- | --- |
