# Supplementary figures and images for: Direct Interaction between TalinB and Rap1 is necessary for adhesion of Dictyostelium cells
Source: BMC Cell Biol. 2016 Jan 7;17:1. doi: 10.1186/s12860-015-0078-0 (PMC4861126; doi:10.1186/s12860-015-0078-0)

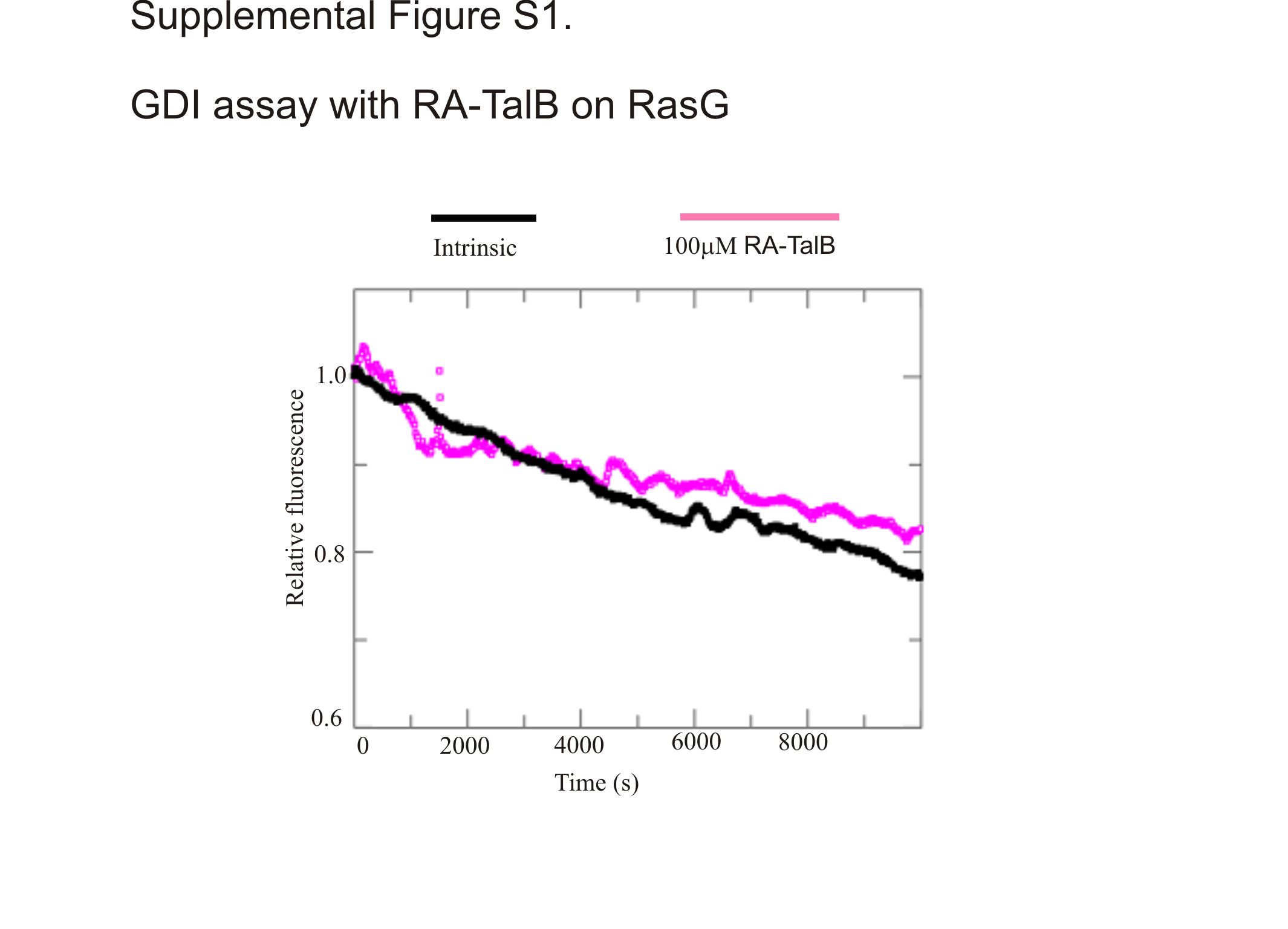

Supplement: Additional file 2: Figure S1. — Dissociation of mGppNHp from RasG in the absence and presence of 100 μM purified RA-TalB. (TIF 430 kb) [file 12860_2015_78_MOESM2_ESM.tif]
